# Rapid Bone Metastasis in Hepatocellular Carcinoma: A Case Report

**DOI:** 10.7759/cureus.90327

**Published:** 2025-08-17

**Authors:** Laraib, Uzma Khalid, Ayesha Khalid

**Affiliations:** 1 Medicine, Quaid-e-Azam Medical College, Bahawalpur, PAK; 2 Medicine and Surgery, Combined Military Hospital (CMH), Lahore, PAK; 3 Medicine, University College of Medicine and Dentistry (UCMD), Lahore, PAK

**Keywords:** bone metastasis, hepatitis c (hcv) infection, hepatocellular carcinoma (hcc), liver cirrhosis, rapid progression

## Abstract

Hepatocellular carcinoma (HCC) is the common malignancy of the liver. It frequently arises in patients with chronic hepatitis infections, with extrahepatic metastases, particularly to bone, typically occurring several months after initial diagnosis. We present a rare case of a 71-year-old female with a 22-year history of hepatitis C infection, and a three-year history of liver cirrhosis. During routine evaluation, a liver ultrasound revealed ill-defined lesions in both liver lobes. Subsequent contrast-enhanced computed tomography (CT) of the liver confirmed the diagnosis of hepatocellular carcinoma. The patient was referred to the oncology department for further management. Remarkably, within three weeks, the patient developed diffuse bone pain, prompting additional investigation. A bone scan revealed widespread skeletal metastases, indicating a rare, rapid progression of HCC. She was initiated on high-dose analgesics and received symptomatic care for other associated symptoms, which provided partial relief. This case highlights the potential for aggressive tumor behavior and the importance of early recognition of atypical metastatic presentations. This also underscores the need for more clinical awareness and prompt diagnostic imaging in similar presentations. Clinicians should maintain a high index of suspicion for bone metastasis in newly diagnosed HCC patients presenting with new-onset bone pain, even in early stages of diagnosis. Early detection and timely palliative intervention are crucial for improving patient outcomes.

## Introduction

Hepatocellular carcinoma (HCC) is the most prevalent type of liver cancer and ranks among the leading causes of mortality globally. In 2022, there were approximately 866,136 new cases and 758,725 deaths worldwide, with an incidence rate of 8.6 per 100,000 and a mortality-to-incidence ratio of 0.86 [1]. Key risk factors for HCC include chronic alcohol consumption, hepatitis B, hepatitis C, and non-alcoholic fatty liver disease. Other, less frequent causes include Wilson's disease, hereditary hemochromatosis, alpha-1 antitrypsin deficiency, primary biliary cirrhosis, and autoimmune hepatitis [2].

Many patients with HCC are asymptomatic, and when symptoms do appear, they are often related to chronic liver disease. These symptoms may include jaundice (yellowing of the skin and eyes), pain in the upper right abdomen, abdominal swelling, weakness, weight loss, fever, and loss of appetite [3]. Furthermore, the rise in diagnoses among asymptomatic individuals is attributed to active surveillance and increased awareness of HCC in high-risk patients, especially those with cirrhosis.

Common extrahepatic sites for liver metastasis include the lungs, lymph nodes, adrenal glands, and bones [4]. The median time from the diagnosis of HCC to the onset of bone metastasis typically ranges from several months to years. However, in rare instances, bone metastases may develop rapidly within weeks [5]. This rapid progression is generally associated with a poor prognosis and can have a significantly negative impact on the patient's quality of life.

## Case presentation

A 71-year-old asymptomatic female with a 22-year history of chronic hepatitis C infection and a known three-year history of hepatitis C-related cirrhosis presented for a routine follow-up evaluation. She had no prior history of hepatocellular carcinoma (HCC) and did not experience any symptoms at that time.

**Figure 1:**
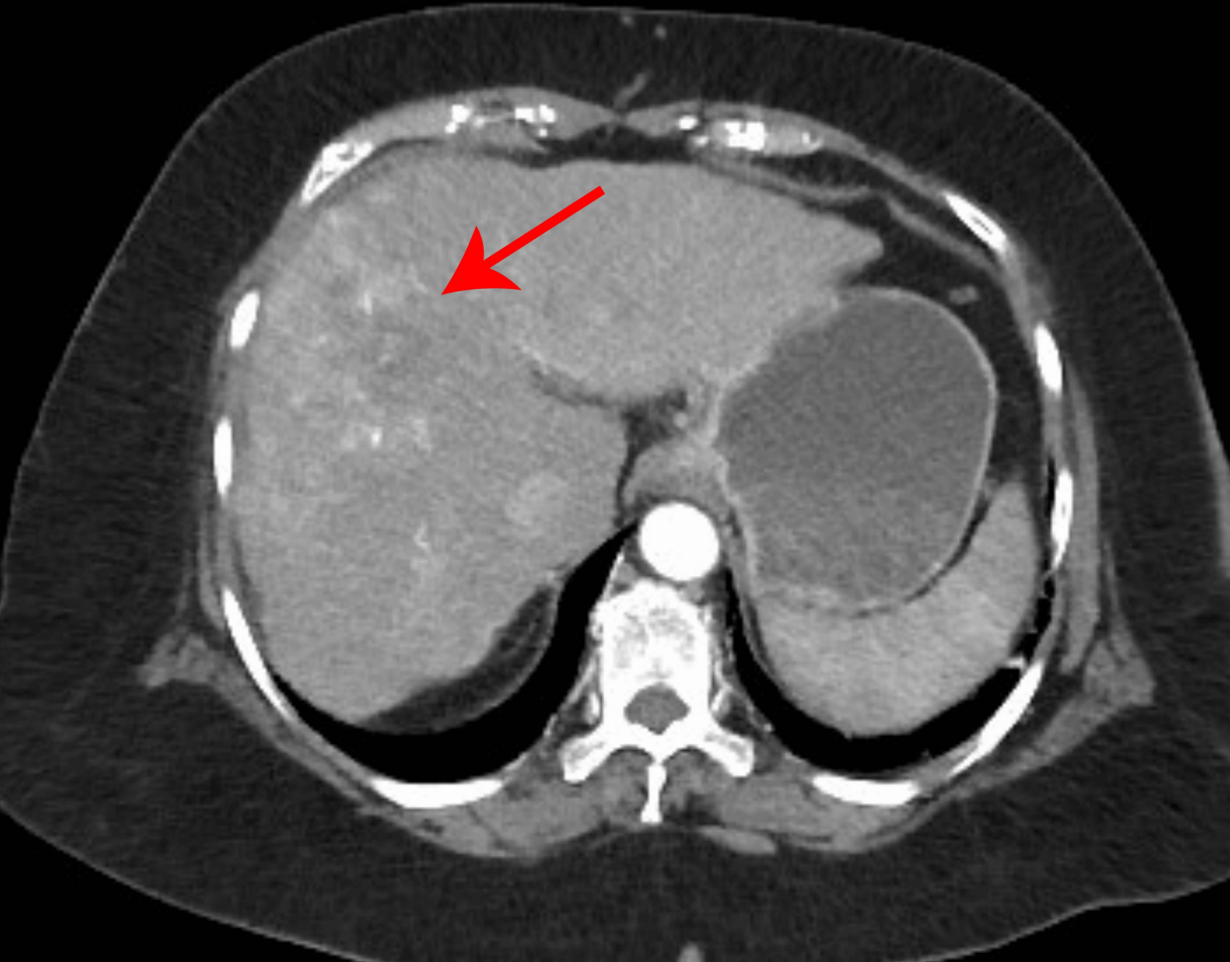
Arterial phase of triphasic CT scan of liver The image shows a contrast-enhanced CT scan of the liver demonstrating multiple variably sized lesions with arterial phase enhancement. These lesions appear brighter than the surrounding normal liver parenchyma. The red arrow highlights one of the hyperenhancing lesions, a typical imaging feature of hepatocellular carcinoma (HCC).

**Figure 2:**
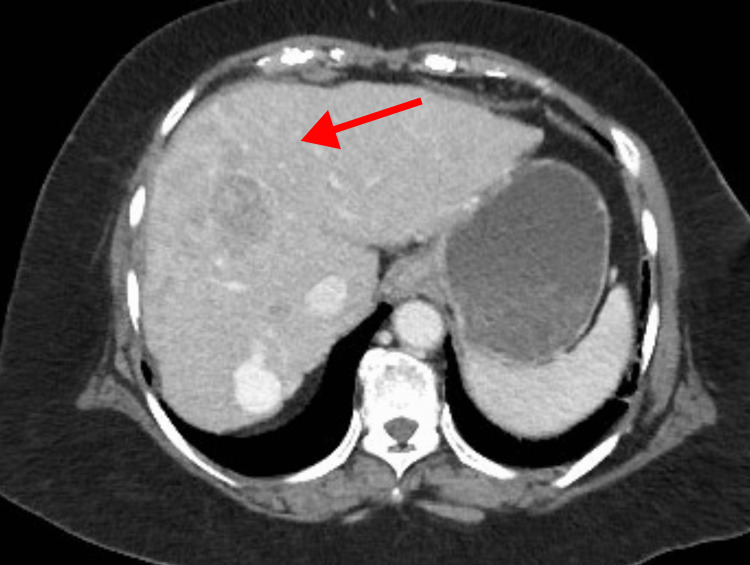
Venous phase of triphasic CT scan The image shows a contrast-enhanced CT scan of the liver in the venous phase, showing lesions exhibiting rapid relative washout. The red arrow highlights a hypodense lesion consistent with the imaging characteristics of hepatocellular carcinoma.

**Figure 3:**
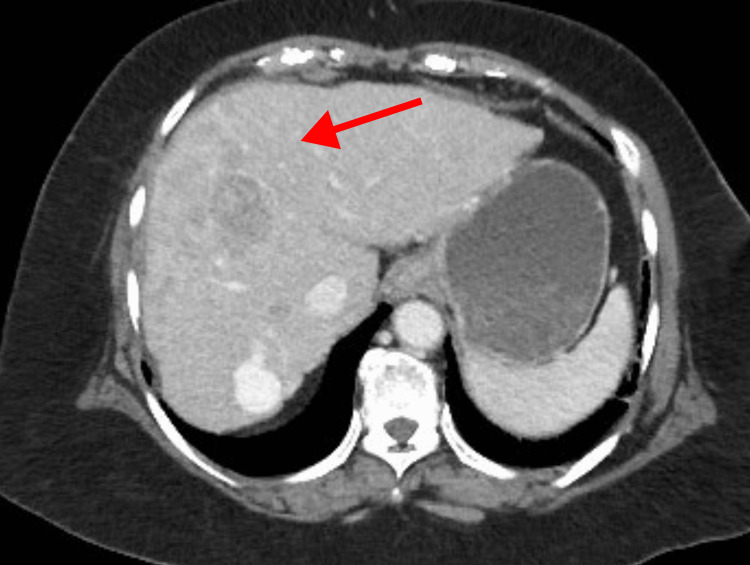
Delayed phase of triphasic CT This image shows a contrast-enhanced CT scan of the liver in the delayed phase. The washout becomes more apparent in this phase, with the tumor appearing significantly hypodense compared to the surrounding liver parenchyma. Rapid washout of contrast, also known as washout, is a specific imaging feature of hepatocellular carcinoma (HCC). The red arrow indicates the hypodense lesion demonstrating these characteristic findings.

During a routine liver ultrasound, ill-defined echogenic lesions with mixed echotexture were observed in the right lobe of the liver, along with hypoechoic masses in both lobes of the liver. These findings prompted further evaluation with a triphasic contrast-enhanced computed tomography (CT) scan of the abdomen. The imaging revealed multifocal poorly marginated lesions in both lobes. These lesions exhibited rapid arterial enhancement, appearing brighter than the surrounding liver tissue (Figure 1). As the contrast washed out of the tumors more quickly than from the normal liver tissue, the HCC lesions appeared hypoattenuating compared to the liver parenchyma in the venous phase (Figure 2). This phenomenon was referred to as "washout". In the delayed phases, the washout effect became more apparent, and the tumors appeared significantly darker than the surrounding liver tissue (Figure 3). The radiological features were consistent with hepatocellular carcinoma in the context of cirrhosis. Importantly, no evidence of extrahepatic disease was detected at the time of the initial diagnosis.

Following the diagnosis, the patient was immediately referred to the oncology department for further assessment and management. However, within three weeks, she developed severe and diffuse bone pain that affected multiple sites, including the pelvis, ribs, spine, shoulders, and long bones. Given the rapid onset and widespread distribution of the pain, a whole-body bone scintigraphy was performed.

**Figure 4:**
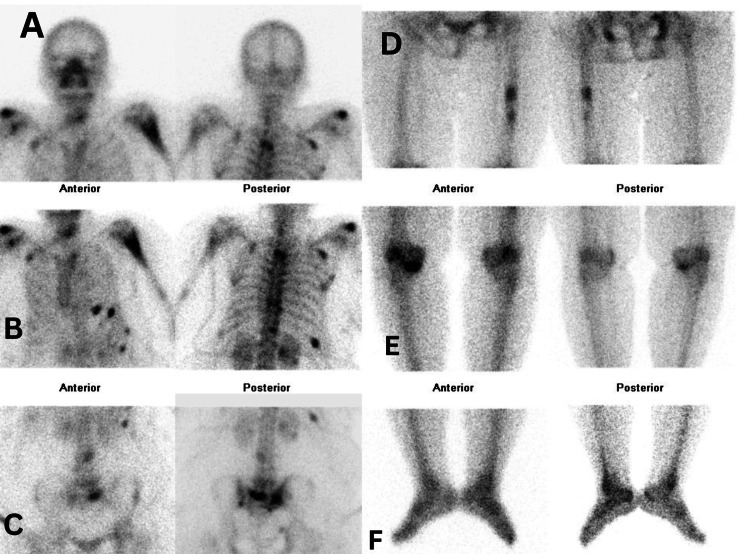
Bone scan showing multiple skeletal metastasis The image shows a body scintigraphy scan demonstrating extensive skeletal metastases. Anterior and posterior views reveal areas of abnormal uptake. (A) skull and shoulder girdle: physiologic uptake without any evidence of focal metastatic lesions; (B) Thoracic spine and ribs: Multiple areas of radiotracer accumulation in the thoracic vertebrae and ribs, consistent with metastatic disease; (C) Lumbar spine and pelvis: Marked uptake in lumbar vertebrae and sacroiliac joints, suggestive of tumor infiltration; (D)Pelvic girdle and femur: Asymmetric distribution in these areas indicates metastatic spread; (E)femoral shaft: Bilateral focal uptake in femoral shafts raises concern for cortical involvement or marrow involvement' (F)Distal lower extremities: physiologic distribution of tracer in distal tibia and feet with no abnormal foci.

The bone scan revealed multiple regions of abnormal radiotracer uptake throughout both the axial and appendicular skeleton (Figure 4). Abnormal uptake was noted in several vertebrae, bilateral ribs, scapulae, shoulders, the left humerus, the left femoral shaft, and bilateral sacroiliac joints. These findings confirmed the presence of multiple skeletal metastases.

Due to the extensive nature of the metastatic disease and the patient's underlying condition, curative and systemic treatment options were deemed unsuitable. Therefore, the patient was started on palliative and supportive care measures. Despite these efforts, her clinical condition progressively deteriorated, ultimately leading to her passing.

## Discussion

Hepatocellular carcinoma (HCC) is a prevalent form of liver cancer, accounting for approximately 80% of all liver cancers. Around 80% of HCC cases occur in Asia and sub-Saharan Africa, primarily due to the high prevalence linked to hepatitis B and C virus infections [6].

Bone metastases in HCC are relatively uncommon, with a reported prevalence ranging from 3% to 20% [7]. These metastases can develop at variable intervals, with some patients experiencing them shortly after diagnosis, while others develop them months or even years later [8]. The unusually rapid progression to skeletal involvement within weeks, as observed in this patient, is rare and suggests a highly aggressive tumor phenotype [9]. 

Bone metastasis in HCC is a recognized but uncommon phenomenon. In a retrospective view, extrahepatic spread at the time of diagnosis is relatively rare, with bone metastasis being less frequent compared to lung or lymph node metastasis. On the other hand, this case illustrates an uncommonly rapid progression of bone metastasis occurring within a short period following initial diagnosis. Previous literature has reported skeletal metastasis as a late finding of advanced disease; our case finding suggests that aggressive bone involvement can occur early in the disease course.

Bone metastasis indicates advanced disease and significantly affects both prognosis and quality of life. In particular, spinal metastases are associated with severe pain, pathological fractures, and neurological deficits. Skeletal-related events such as fractures, spinal cord compression, and hypercalcemia are also common. HCC-related bone metastases drastically reduce median survival, often to just a few months. The resulting pain, functional limitation, and potential for neurological deficits severely impact a patient's quality of life [10].

Moreover, treatment for bone metastases often requires surgery, radiation therapy, and pain management, which can lead to increased healthcare costs and resource utilization. Given the poor prognosis associated with HCC-related bone metastases, early detection through routine screening is crucial for improving patient outcomes.

## Conclusions

Rapid bone metastasis in hepatocellular carcinoma is rare but possible, as demonstrated by this case. Clinicians should remain cautious about metastatic disease in HCC patients with new or worsening bone pain, regardless of the time since diagnosis. Prompt imaging and multidisciplinary management can improve symptom control and patient outcomes. Metastases occurring on the background of decompensated cirrhosis can pose significant challenges to patient management, as symptoms may overlap and exacerbate each other. A well-planned, strategic approach is essential to ensure optimal comfort and care for such patients.
